# Advances in Pluripotent Stem Cells: History, Mechanisms, Technologies, and Applications

**DOI:** 10.1007/s12015-019-09935-x

**Published:** 2019-11-23

**Authors:** Gele Liu, Brian T. David, Matthew Trawczynski, Richard G. Fessler

**Affiliations:** Department of Neurosurgery, Rush University Medical College, 1725 W. Harrison St., Suite 855, Chicago, IL 60612 USA

**Keywords:** Advances, Stem Cells, Technologies, Applications

## Abstract

Over the past 20 years, and particularly in the last decade, significant developmental milestones have driven basic, translational, and clinical advances in the field of stem cell and regenerative medicine. In this article, we provide a systemic overview of the major recent discoveries in this exciting and rapidly developing field. We begin by discussing experimental advances in the generation and differentiation of pluripotent stem cells (PSCs), next moving to the maintenance of stem cells in different culture types, and finishing with a discussion of three-dimensional (3D) cell technology and future stem cell applications. Specifically, we highlight the following crucial domains: 1) sources of pluripotent cells; 2) next-generation *in vivo* direct reprogramming technology*;* 3) cell types derived from PSCs and the influence of genetic memory; 4) induction of pluripotency with genomic modifications; 5) construction of vectors with reprogramming factor combinations; 6) enhancing pluripotency with small molecules and genetic signaling pathways; 7) induction of cell reprogramming by RNA signaling; 8) induction and enhancement of pluripotency with chemicals; 9) maintenance of pluripotency and genomic stability in induced pluripotent stem cells (iPSCs); 10) feeder-free and xenon-free culture environments; 11) biomaterial applications in stem cell biology; 12) three-dimensional (3D) cell technology; 13) 3D bioprinting; 14) downstream stem cell applications; and 15) current ethical issues in stem cell and regenerative medicine. This review, encompassing the fundamental concepts of regenerative medicine, is intended to provide a comprehensive portrait of important progress in stem cell research and development. Innovative technologies and real-world applications are emphasized for readers interested in the exciting, promising, and challenging field of stem cells and those seeking guidance in planning future research direction.

## Introduction

**Fig. 1.**
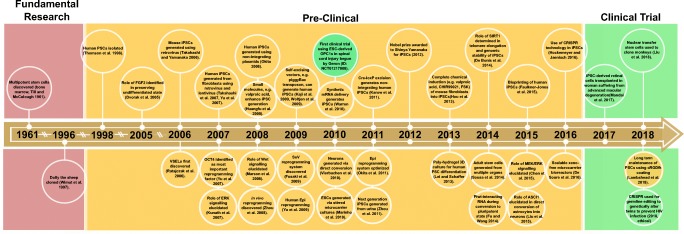
The timeline of major scientific advances during the history of stem cell research. Multipotent stem cells were first discovered in 1961, representing the initial breakthrough in stem cell and regenerative medicine. Dolly the sheep was cloned in 1997. The transition from fundamental research, to pre-clinical research, and finally to clinical trials is driven by many discoveries and milestones. Many advances in reprogramming factor combinations, experimental methods, and the elucidation of signaling pathways have recently contributed to the first clinical trials for retinal cell transplants and spinal cord transplants. Red shading represents fundamental research, yellow shading represents pre-clinical work, and green shading represents clinical trials.

Historically, many key milestones have driven progress in the field of stem cell research [Fig. 1] More than half a century ago, in 1961, the first stem cells were described by Drs. James A. Till and Ernest A. McCulloch at the University of Toronto in Canada [1]. They found that stem cells derived from mouse bone marrow cells had the ability to differentiate into a variety of cell types, and were thus called pluripotent stem cells (PSCs). Several decades later, in 1996, Dolly the sheep was cloned by Keith Campbell, Ian Wilmut, and colleagues at the Roslin Institute of the University of Edinburgh in Scotland, demonstrating the validity of the somatic cell nuclear transfer (SCNT) [2]. Then, in 1998, the first human embryonic stem cells (hESCs) were isolated by James Thomson in the USA [3]. In 2006, induced PSCs (iPSCs) were derived from reprogrammed adult somatic cells with just four basic transcription factors, reduced from 24 factors [4,5]. In 2012, Shinya Yamanaka (Kyoto University, Japan and Gladstone Institutes, USA) and John Gurdon (Gurdon Institute, Cambridge, UK) were co-recipients of the Nobel Prize for Physiology or Medicine for their discovery that mature cells could be reprogrammed into a pluripotent state [6]. Researchers have since detected innate adult stem cells within several organs [7–9]. To date, five basic categories of stem cells have been put forward following our systematic review of stem cell research: embryonic stem cells (ESCs), very small embryonic-like stem cells (VSELs), nuclear transfer stem cells (NTSCs), reprogrammed stem cells (RSCs), and adult stem cells (ASCs) (see Table 1). Only NTSCs have been used to generate a complete organism: monkeys were grown from NTSCs in China in 2018 [10]. On the other hand, ESCs, iPSCs, and adult stem cells have only been used to generate tissues and organs. In recent years, and especially in the last decade, stem cell research has blossomed into an exciting and promising field. Stem cells, especially ESCs and iPSCs have shown great application promise in four major fields: regenerative and transplant medicine [11,12]; disease modeling [13,14]; drug discovery screening [15,16]; and human developmental biology [17] [18],. Thus, the evolution of regenerative medicine continues, from the early first descriptions of stem cells to their expanding clinical applications at present.

**Table 1:** Five Basic Categories of Stem Cells

	**Embryonic stem cells (ESCs)**	**Very Small Embryonic-Like Stem Cells (VSELs)**	**Nuclear transfer stem cells (NTSCs)**	**Reprogramming Stem Cells** **(RSCs)**	**Adult stem cells** **(ASCs)**
Definition	Pluripotent stem cells derived from the inner cell mass of a blastocyst (embryo)	Pluripotent stem cells derived from adult tissues	One new single cell is produced by the transplantation of the donor nucleus into an enucleated oocyte of a donor egg. Reprogramming occurs to form blastocyst.	Pluripotent stem cells generated by reprogramming adult cells. Derived by applying manual laboratory methods to reprogram adult cells (except SCNT). RSCs include iPSCs and direct reprogramming stem cells.	A type of cell in close proximity to rich, nutrient-full microenvironment such as vessels, bone marrow, or organs (heart and brain, etc) in the mature or adult organism; they are able to respond to tissue-specific stimulation to produce stem cells.
Development stage	Early-stage pre-implantation embryo; Human embryos generate the blastocyst (50–150 cells) 4–5 days post-fertilization	Early developmental stem cell mass in adult tissues	One blastocyst is about 100 cells at early stage embryo	Early embryonic stage that can be single or multiple cells; or specific tissue-lineage cells	Mature stem cells from adult cells (such as umbilical cord blood cells) or adult organs such as heart and brain
Morphology	Blastocyst (multiple cells); uncertain shape without resembling any specific cell	Appears similar to inner cells of blastocyst	Complete single cell; generalized shape without resembling any specific cell	iPSCs: single or multiple cells as blastocyst, generalized, uncertain shape without looking like any specific cell; Specific tissue-lineage cells are similar.	Complete single or multiple cells; the shape looks like mature cells of a particular organ system.
First event	hESCs were isolated in US in 1998	VSELs were isolated in the US in 2006	Dolly the sheep was cloned in the UK in 1996	Four Yamanaka factors (Oct4, Sox2, Klf4, and cMyc) elucidated in Japan in 2006	Bone marrow cells in Canada in 1961
Example of representative cells or organism for medical applications	hESC-derived oligodendrocyte progenitor cells (OPCs): AST-OPC1s used in the first clinical trial in the US in 2010	An alternative to monopotent tissue-committed stem cells in adults	Monkeys in China in 2018	Reprogramming mature cells such as peripheral blood cells, fibroblasts, keratinocytes, and urine cells	Umbilical cord blood cells, bone marrow cells, and endogenous stem cells, such as in heart, brain, and spinal cord
Function	Totipotent embryonic stem cells in morula: able to develop into any type of cell	Totipotency of VSELs remains unclear; but cells can differentiate into mesenchymal stem cells, hemangioblasts, and endothelial progenitor cells, as well as tissue-committed stem cells	Single cell generates a whole organism	Develops into any type of cell	Develops into cells of the same systemic type
Final products by different competencies	To produce any types of cells, tissues, and organs	Potential to produce various cells across germ layers in adult animals or humans	To generate a living organism	To produce any types of cells, tissues, and organs, like ESCs	To produce cells, tissues, and organs in the same genetic lineage
Applications	Four major fields: regenerative and transplant medicine, disease modeling, drug discovery screening, and human developmental biology	Four major fields: regenerative and transplant medicine, disease modeling, drug discovery screening, and human developmental biology	Four major fields: regenerative and transplant medicine, disease modeling, drug discovery screening, and human developmental biology	Four major fields: regenerative and transplant medicine, disease modeling, drug discovery screening, and human developmental biology	Four major fields: regenerative and transplant medicine, disease modeling, drug discovery screening, and human developmental biology
To obtain	Harvest from unviable embryo, surgery, abortion	Invasive surgery or noninvasive collection	Surgery to get single nucleus donor and egg donor	Invasive surgery or noninvasive collection	Invasive surgery to obtain or injection of growth factors or small molecular chemicals into certain tissues for stimulation of endogenous stem cells
Major issues	Destruction/abortion of embryo; immune rejection, and depletion of cell resources	To determine overall properties and functions	May be abused in human cloning; high requirements for technology, facility, and finance	Genomic instability; can have low efficacy	Invasive surgery, immune rejection (if non-autologous donor), contamination, and infection, as well as cannot naturally cross genetic barriers to differentiate into other lineage
Future	Phased out over time	Significant promise	Limited development	Significant promise	Significant promise

As iPSC reprogramming technology is still relatively new, challenges remain – especially with respect to cell proliferation and differentiation. Therefore, in this review, we systematically review the following methodological topics: induction of pluripotency by genomic modifications; the construction of novel vectors in combination with reprogramming factors; promotion of iPSC pluripotency with small molecules and genetic signaling pathways; induction and enhancement of reprogramming with microRNAs; induction and enhancement of iPSC pluripotency with chemicals; generation of specific differentiated cell types; and maintenance of iPSC pluripotency and genomic stability. Ultimately, these topics are crucial for maximizing the efficacy of iPSC generation and differentiation in preparation for clinical translation. We also consider advances in cell culture, namely feeder-free culture, xeno-free media, and various biomaterial-augmented techniques. Further, we include discussions of three-dimensional (3D) cellular and bioprinting technologies, PSC resources, and second-generation direct cellular reprogramming *in vivo*. Finally, long-term stem cell research and clinical goals are considered.

The overall purpose of this article is to provide a synopsis of significant historical and recent research advancements in stem cell and regenerative medicine. Although a detailed presentation of all relevant stem cell data and subtopics would be beyond the scope of this article, we do provide guidance to help readers identify resources for deeper study.

## Sources of pluripotent stem cells

PSCs are characterized by the properties of self-renewal and potency, wherein the former refers to the cell’s ability to proliferate and the latter refers to the cell’s ability to differentiate into specialized cell types derived from one of three primary germ layers: ectoderm, endoderm, or mesoderm [19]. Aoi (2016) summarized three *in vivo* assays to assess the potency of pluripotent stem cells in mouse models [20]. The first model is the teratoma formation assay, which is used to evaluate the spontaneous generation of differentiated tissues from the three germ layers after the transplantation of cells into immunocompromised mice. The second model is the chimera formation assay, which tests whether stem cells contribute to development by injecting these cells into diploid early embryos (2N blastocysts). Chimeras are then bred, and other assay endpoints include when the donor cells have germline transmission capacity, generate functional gametes, and retain chromosomal integrity with functional pluripotency. The third model is the tetraploid (4N) complementation assay, which is used to determine the capacity of the tested pluripotent cells within an entire organism. After injecting cells into 4N embryos (4N blastocysts), the stages of growth are monitored for extra-embryonic lineages as a result of the transplanted stem cells and not the embryo itself.

The five basic stem cell types are ESCs, VSELs, iPSCs, NTSCs, and adult stem cells. Each cell type may be harvested or generated from various sources (see Table 1). The features of each cell types are described as follows:

1. **Embryonic Stem Cells.** Human ESCs (hESCs) are harvested from early-stage blastocysts (4~5 days postfertilization) by destroying the source blastocyst or by harvesting later stage (3 month gestational age or less) tissues. hESCs are the first stem cells to have been applied in research applications, especially, they are still commonly used in the clinical trials at present (https://clinicaltrials.gov/).
2. Recently, one novel type of pluripotent stem cell - Very Small Embryonic-Like Stem Cells (VSELs) – has shown promise [21]. VSELs were identified in 2006 by Ratajczak et al. [22], and over 20 independent laboratories have since confirmed their existance [21,23–25]. This being said, other groups have questioned their existence [26]. These cells are small and early development stem cells in adult tissues, which express pluripotency markers, and according to their primitive morphology and gene expression profile, are termed VSELs [27]. Regarding its morphology, VSELs are small cells, corresponding to the cells in the inner cell mass of the blastocyst, which are about 3 to 5 μm in mice and around 5 to 7 μm in humans (slightly smaller than red blood cells). For gene expression profile, VSELs express some ESCs markers, such as *SSEA*, nuclear *Oct-4A*, *Nanog*, and *Rex1* [21]. VSELs also express several markers for migrating primordial germ cells (PGCs), such as Stella and Fragilis [21]. Additionally, VSEL single-cell cDNA libraries shown murine bone marrow-isolated biomarkers such as very small Sca-1+lin-CD45-cells [28]. Thus, the developmental origin of VSELs may be associated with germline deposits in developing organs during embryogenesis [27]. Ratajczak [21] (2019) proposed a VSEL developmental and functional model. According to this model, VSELs originated from primordial germ cells (PGCs) and further differentiated into three potential fates - mesenchymal stem cells (MSCs), hemangioblasts [two subtypes of hematopoietic stem cells including (HSCs) and endothelial progenitor cells (EPCs)], and tissue-committed stem cells (TCSCs). Thus, VSELs, as a pluripotent stem cell, may hold a potential advantage of being able to differentiate across germ layers in adult animals or human subjects. Such cells may function as an alternative to monopotent tissue-committed stem cells in adults [27]. In addition, VSELs may overcome several problems of ESCs (ethical controversies) and iPSCs (teratoma formation) for future stem cell studies and clinical applications.
3. **Nuclear Transfer Stem Cells.** Originally discovered in 1996, the somatic cell nuclear transfer (SCNT) technique has gradually evolved and can now generate NTSCs. SCNT begins by first implanting a donor nucleus (i.e. nucleus donor) from another fully differentiated somatic cell (e.g. fibroblast) into an enucleated oocyte (i.e. cytoplasmic donor or egg donor with nucleus removed). Then, the new host egg cell triggers the genetic reprogramming of the donor nucleus. Subsequently, numerous mitotic divisions of this single cell in culture develop a blastocyst, which is about 100 cells at early-stage embryo. The end result generates an organism with almost identical DNA to the original organism – a clone of the nuclear donor. Such a nucleus donor cloning is a dominated genotypes and phenotypes, while the cytoplasmic donor or egg donor has some genotypes and phenotypes in this new entire living organism as well. This process can produce both therapeutic and reproductive cloning. In July 1996, Dolly the Sheep was the first successful reproductive clone of a mammal, which was performed in Scotland, United Kingdom [29] [30] [31],., Thus far, some two dozen other species have been cloned [32]. Recently, in January of 2018, Chinese scientists in Shanghai announced the successful use of fetal fibroblasts to clone two female macaque monkeys by SCNT [10], thus creating the first primates to be cloned by SCNT.

Creating cloned primates could revolutionize human disease research [32]. Genetically uniform non-human primates may be useful animal models for primate biology and biomedical research. Such animal models could be used to investigate disease mechanisms and drug targets, obviating the confounding factor of genetic variation, thereby reducing the number of laboratory animals needed [32]. The technology could also be combined with CRISPR-Cas9 genomic-editing to create genetically engineered primate models of human disorders, such as Parkinson disease (PD) and various cancers. Pharmaceutical companies have signaled a high demand for cloned monkeys to use in drug testing [32]. Enthused by the potential of this prospect, the city of Shanghai has prioritized funding for the establishment of an International Primate Research Center that can produce cloned research animals for use internationally [32]. Relative to other stem cell approaches, SCNT is unique in that it can generate an entire living body rather than sheets of cells, tissues, and pieces of organs, which can be created with ESC and iPSC protocols. From the perspective of biophysiological function, SCNT thus has advantages over ESCs and iPSCs for basic research and clinical application.

(4). **Reprogrammed Stem Cells.** Since 2006 when Yamanaka and colleagues first generated iPSCs, reprogramming technologies in general have significantly progressed. This is especially true with respect to direct reprogramming methods *in vitro* and *in vivo* to produce specific tissue-lineages by using lineage-restricted transcription factors, RNA signal modifications, and small molecules or chemicals. These direct approaches skips the iPSCs step yielding more precise cells, such as induced neural progenitor cells (iNPCs), which are closer to the target cell lineage, such as neural cells and subsequent motor neurons. Thus, reprogrammed stem cells (RSCs) are derived from by applying any manual laboratory methods to reprogram genetic signals of the primary cells, but they do not include the SCNT technique.

To overcome the ethical and immunogenic challenges associated with hESCs, iPSCs have emerged as a promising alternative. This is because iPSCs are derived from adult somatic tissues, and hiPSC sources, such as blood, skin, and urine, are plentiful. In addition, because hiPSCs can be harvested from individual patients, immune rejection can be avoided when they are transplanted autologously (self-donor). Thus, hiPSCs have extraordinary potential for personalized medicine. A variety of iPSC sources exist. In theory, almost any mature cell type in the human body, including umbilical cord blood cells, bone marrow cells, peripheral blood cells, fibroblasts, keratinocytes, and even cells in urine can be reprogrammed into iPSCs and then be differentiated into tissue-specific cells of desired lineages [33] [34] [35],., To be clear, mature (a.k.a. “adult”) stem cells refer to the differentiated state of the cells themselves, not the maturity (or adult status) of the body from which they were harvested. Umbilical cord blood or bone marrow stem cells are considered “ready-to-use” in that they can be employed directly for transplantation without reprogramming. Adult stem cells will be discussed in more details in the following section. Non-autologous (i.e., non-self) stem cells carry an inherent risk of immune rejection. Easily accessible tissues for autologous stem cell harvesting include skin, hair, and urine. To avoid any further discomfort or risk in patients - especially medically fragile patients who have suffered traumatic medical events such as a heart attack or spinal cord injury (SCI) – urine is a noninvasive stem cell source. Although cells harvested from urine have not yet received substantial research and attention, it is our view that they are a highly promising stem cell source which warrant further research.

Noninvasive, reproducible, simple, and easily accessible mature somatic cell sources and harvesting protocols are needed for development of directed iPSC differentiation for broader clinical use. In addition to these features, urine samples provide an unlimited autologous cell source, and cells obtained from urine samples have robust reprogramming characteristics. Urine is a relatively untapped source of autologous MSCs [36]. A method for obtaining hiPSCs from renal tubular cells present in urine was described by Zhou and colleagues in July 2011, with a more detailed protocol for obtaining exfoliated renal epithelial cells being published by the same group one year later [37]. The latter method, which requires only a 30-ml sample of urine, is simple, relatively fast, cost-effective, and universal (applicable to patients of all ages, genders, and racial/ethnic backgrounds). The total procedure involves just 2 weeks of cell culturing and 3-4 weeks of reprogramming. It produces high iPSC yields with excellent differentiation potential. Urine-derived iPSCs collected from 200 mL clean midstream urine samples via the Sendai virus delivery system showed a normal karyotype and exhibited the potential to differentiate into three germ layers in a teratoma assay [38]. In addition, Zhang and colleagues reported that a subpopulation of cells isolated from urine had progenitor cell features, including cell-surface expression of c-Kit, SSEA4, CD105, CD73, CD91, CD133, and CD44, markers that can be used to distinguish among bladder cell lineages (e.g. urothelial, smooth muscle, endothelial and interstitial) [39]. Thus, these cells could serve as an alternative cell source for urinary tract tissue engineering and reconstruction. Similarly, upper urinary tract cells have been reported to possess expansion and differentiation capabilities for forming urothelial and myogenic cells, which could potentially be used for bladder tissue engineering in patients needing cystoplasty [40]. Unfortunately, neither of these studies used an iPSC stage before differentiation; they collected urothelial and myogenic cells only. Importantly, however, an hiPSC development approach for urine-derived cells was described for stored iPSCs under feeder-free, virus-free, serum-free conditions without use of the oncogene *c-Myc* [41]. This bank produced 93 hiPSC lines from 20 genetically diverse donors.

Urine samples have been shown to be a good alternative option for harvesting iPSCs to be differentiated into different cell subtypes across various systems. In the cardiovascular system, urine cell-derived functional cardiomyocytes were shown to generate action potentials, both *in vitro* and *in vivo*, following differentiation of reprogrammed iPSCs by lentiviral-vector gene transduction [42]. With respect to metabolic diseases, iPSCs were generated from urine cells from one patient with a mitochondrial DNA mutation [43]. In the endocrine system, human urine-derived stem cells facilitated diabetic wound repair by promoting angiogenesis [44]. Additionally, in a neuroendocrine application, cells obtained from the urine of patients with multiple endocrine neoplasia type 1 syndrome (MEN1) were used to generate iPSCs with non-integrated episomal plasmids carrying *Oct4*, *Sox2*, *Klf4,* and miR-302-367 without using *c-Myc* [45] [46],. In the field of psychiatry, an iPSC line derived from a urine sample of a patient with obsessive-compulsive disorder was produced with an integration-free CytoTune®-iPS 2.0 Sendai reprogramming kit [47].

Applications of iPSC technology to the nervous system also exist. Integration-free neural progenitor cells generated by reprogramming of epithelial-like cells from human urine can be differentiated into multiple functional neuronal and glial subtypes *in vitro* [48]. Recent data obtained in experimental animal models showed that reprogrammed integration-free iPSCs derived from human neural progenitors collected from urine differentiated into neurons and glia within 8 weeks of being transplanted into contused mouse thoracic spinal cords, though the study lacked functional data with respect to SCI recovery and included the oncogene *c-Myc* in its reprogramming protocol [49].

Recent experiments indicated that urine-derived iPSCs are a promising resource for motor neuron disease modeling and cell therapy development [50,51]. In addition, urine cells from a patient with spinocerebellar ataxia type 3 (autosomal dominant inherited neurodegenerative disease) were transformed into iPSCs with a SeV delivery system, providing a robust platform for further study of this disease’s pathogenesis and its susceptibility to pharmacotherapy as well as gene therapy [52]. Recently, iPSCs generated from urine-derived cells from a patient with spinal muscular atrophy with an Epi reprogramming vector (c-Myc-free and non-integrating) combined with CRISPR technology were used to correct the disease-causing mutation at the iPSC level, and these cells were then were developed into motor neurons [53]. Such a protocol may eventually lead to gene therapy for spinal muscular atrophy.

The aforementioned studies have demonstrated that urine samples represent an extremely valuable resource for cells with high reprogramming efficiency. Additional evidence is needed with respect to the efficiency of such cells for producing various subtypes of nervous system cells (e.g., subtypes of oligodendrocytes, astrocytes, sensory neurons, and motor neurons). Such cells derived from urine cells would be expected to have a genetic or epigenetic memory of their primary genotype-phenotype, which may prevent the efficacy of transformation. Thus, challenges remain. Physiological functional studies will be critical for bringing urine sample-derived stem cells into clinical practice.

(5). **Adult Stem Cells.** When first discovered, adult stem cells generated significant excitement surround their translational applications, however, questions remain about their clinical utility. Adult stem cells harvested from specific organs, such as the brain, spinal cord, or heart, may offer a novel direction for cell therapy. Characterization of stem cells in adult organs has suggested that their survival, quiescence, and activation depend on precise signals in their microenvironment [54]. They often appear to have the capacity to recognize damaged sites and dying cell types, regenerating only missing cells. Tissue-resident adult stem/progenitor cells are potentially easily accessible sources for cell therapy. These cells have a high self-renewal ability and multilineage differentiation potential to reconstitute damaged tissues without immune rejection. In the other hand, adult stem cells harvested from mature tissues may be reprogrammed into iPSCs, as discussed above. Exogenous biological small molecules may also be used to stimulate endogenous cells *in situ* to grow and differentiate into specific cell types.

The most important subcategory of adult stem cells is MSCs***.*** In particular, these are the most widely used adult stem cells at present. Although MSCs were isolated initially from bone marrow, other adult tissues sources have also been identified [7]. The major sources of human MSCs are umbilical cord blood, bone marrow, adipose-derived, placental and amniotic fluid, and menstrual blood. Umbilical cord blood, which can only be collected at birth, has several practical considerations, such as banking safety, contamination, and identity and quality issues after long-term storage. There are several standardized operating procedures for obtaining clinically useful cord blood for future use to benefit infant donors [55], such as adhering to informed consent policies, financial disclosures, conflict-of-interest policies, and others [56] [57,58],. Stem cells from bone marrow has been widely studied *in vitro* and in animal models, but clinical trials have shown only limited effectiveness.

The exciting discovery of adult stem/progenitor cells in the brain and heart [59] has inspired hope that such endogenous stem cells may someday be used to repair tissues damaged in myocardial infarction and stroke. To use these MSCs, they must be identifiable with biomarkers. For example, the International Society for Cellular Therapy recommends identifying hMSCs with immunopositivity for CD105, CD73, and CD90 surface antigens (expressed by ≥95% of such cells), combined with immunonegativity for CD45, CD34, CD14 or CD11b, CD79a or CD19, and human leukocyte antigen–DR isotype (≤2% positivity among hMSCs) [7].

As mentioned above, signal transduction pathway stimulation can improve transformation efficacy for both exogenously and endogenously sourced stem cells. Both ESC and iPSC culture systems can be applied for *in vitro* generation of desired cells for transplantation into patients. Alternatively, small biomolecules (e.g. growth factors) may be injected into damaged living tissues to promote differentiation of endogenous adult stem cells into desired cell types, such as motor neurons, sensory neurons, oligodendrocytes, and astrocytes in neural tissue damaged by SCI or stroke. Although this latter method may seem simple in principle, such approaches have not yet been validated outside of animal models for clinical applications. Indeed, further evidence is needed to clarify the relative feasibility and efficacy of these two approaches. It is possible that both approaches may be combined to further optimize outcomes [60].

## Second generation: Direct cellular reprogramming *in vivo*

Here we explore further the promise of second-generation cellular reprogramming by way of direct *in vivo* approaches, which may overcome critical challenges associates with *in vitro* systems such as shifting cell arrangements and functions, contamination, and time-intensive processing [61]. The fundamental principles are similar to those in first generation *in vitro* approaches, except that all protocols are carried out entirely within living animals in native target tissues (e.g. mouse brain, heart). This approach relies on the native microenvironment to produce natural products and obtain *in situ* recovery of locally degenerated and damaged tissues.

In direct *in vivo* cellular reprogramming, lineage-restricted transcription factors and microRNAs are used to reprogram resident support cells to generate desired cell types. The reprogramming differs from those used *in vitro* because it is more universal and encompasses early-stage reprogramming factors, such as OSKM, which are able in theory to differentiate stem cells into any type of cell in the body. Lineage-restricted transcription factors and microRNAs have the potential to reprogram local somatic cells to differentiate into specific types of cells without an intermediary stem/progenitor cell stage. The mechanisms mediating such reprogramming is unclear, but are likely driven by forces involving cellular memory and the native microenvironment.

*In vivo* somatic cell reprogramming research has made substantial progress recently, especially for cardiomyocyte and neuronal fates. In 2008, Zhou et al. reported on the *in vivo* reprogramming of pancreatic exocrine cells into beta cells with the transcription factors *NGN3, PDX1*, and *MAFA* [62]. The Zhou study paradigm provides a potential blueprint for directing cell reprogramming without reversion to a PSC state. In addition, use of the transcription factors *FOXa3, GATA4, HNF1a*, and *HNF4a* generated hepatocyte-like cells directly from myofibroblasts in fibrotic mouse livers and reduced liver fibrosis *in vivo* [63], suggesting this approach may lead to treatment for chronic liver disease.

In the cardiovascular system, mouse cardiac fibroblasts have been reprogrammed using cardiac developmental transcription factor genes - namely, *Gata4*, *Mef2c*, and *Tbx5* with [64] or without [65] HAND^-2^. These were transplanted and subsequently induced the development of cardiomyocyte-like cells. These were electrically incorporated into myocardial tissue and used to improve cardiac function in a cardiac injury model. It is hoped that this line of research may lead to clinical protocols to utilize the endogenous bulky pool of fibroblasts within the heart for targeted cell therapy.

In the nervous system, endogenous mouse astrocytes can be converted directly into neurons (neural nuclei protein immunopositive) *in situ* with transplanted human cells or endogenous mouse cells as starting cells. The neural conversion genes include *Ascl1*, *Brn2a*, and *Myt1l* [66]. In fact, a single transcription factor gene, *Ascl1*, is sufficient to convert brain astrocytes into functional neurons *in vivo* [67]. *Ascl1* has been used *in vivo* to reprogram retinal Müller glia toward a neuronal fate [68]. In the adult mouse brain, *Sox2* was sufficient to reprogram resident astrocytes into proliferative induced adult neuroblasts, which went on to develop into electrophysiologically mature neurons that functionally integrated into local neural networks in the presence of brain-derived neurotrophic factor, noggin, or when the mice were treated with a histone deacetylase inhibitor [60]. Interestingly, *Sox2* has also been used to reprogram pericytes in the brain into induced neurons [69]. These results demonstrate that adult astrocytes have the potential for extraordinary plasticity *in vivo*. Notably, the latter experiment demonstrated the methodological feasibility of both reprogramming and injection to induce endogenous cells to differentiate into a specific type of cell *in vivo*.

## Types of differentiated cells and genetic memory

Stem cells can be transformed into specific types of cells via reprogramming and subsequent differentiation. There are three critical aspects of ongoing research into stem cell development and differentiation: differences between iPSCs and ESCs, genetic “memory” of cells/tissues, and direct working systems *in vitro* or *in vivo*.

Direct comparisons of neural-differentiation capacity between human iPSCs and ESCs have suggested that human iPSCs generate neuroepithelia and functionally appropriate neuronal types, similar to the outcomes obtained with hESCs under the same conditions [70]. Relative to ESCs, however, iPSCs, were found to be less efficient and to exhibit greater variability, deficiencies that could be improved with culturing technique alterations [70]. Some have found that particular iPSC lines may be epigenetically unique and inclined to generate cells of a certain lineage [70]. Once a cell type has fully matured, such as an adult fibroblast, iPSCs derived from this cell type may carry a genetic "memory" of the primary cell type, and it can be difficult to “reprogram away” completely [71]. Epigenetic memory may also be responsible for the lineage-specific bias of some hiPSCs [72]. It remains to be clarified how this genetic memory diverges among different cell types and tissues.

Specific types of desired cells may also be obtained directly *in vitro* or *in vivo* without a stem cell reprogramming process. For example, after screening a pool of nineteen candidate genes, the combination of three factors genes, *Ascl1*, *Brn2*, and *Myt1l*, was shown to be sufficient to induce rapid transformation of *in vitro* mouse ESCs and postnatal fibroblasts into functional neurons, which not only express multiple neuron-specific proteins but also produce action potentials and form functional synapses [73]. Direct *in vivo* approaches for producing iPSCs are discussed in more detail above.

## Inducing pluripotency with genomic modifications

**Fig. 2.**
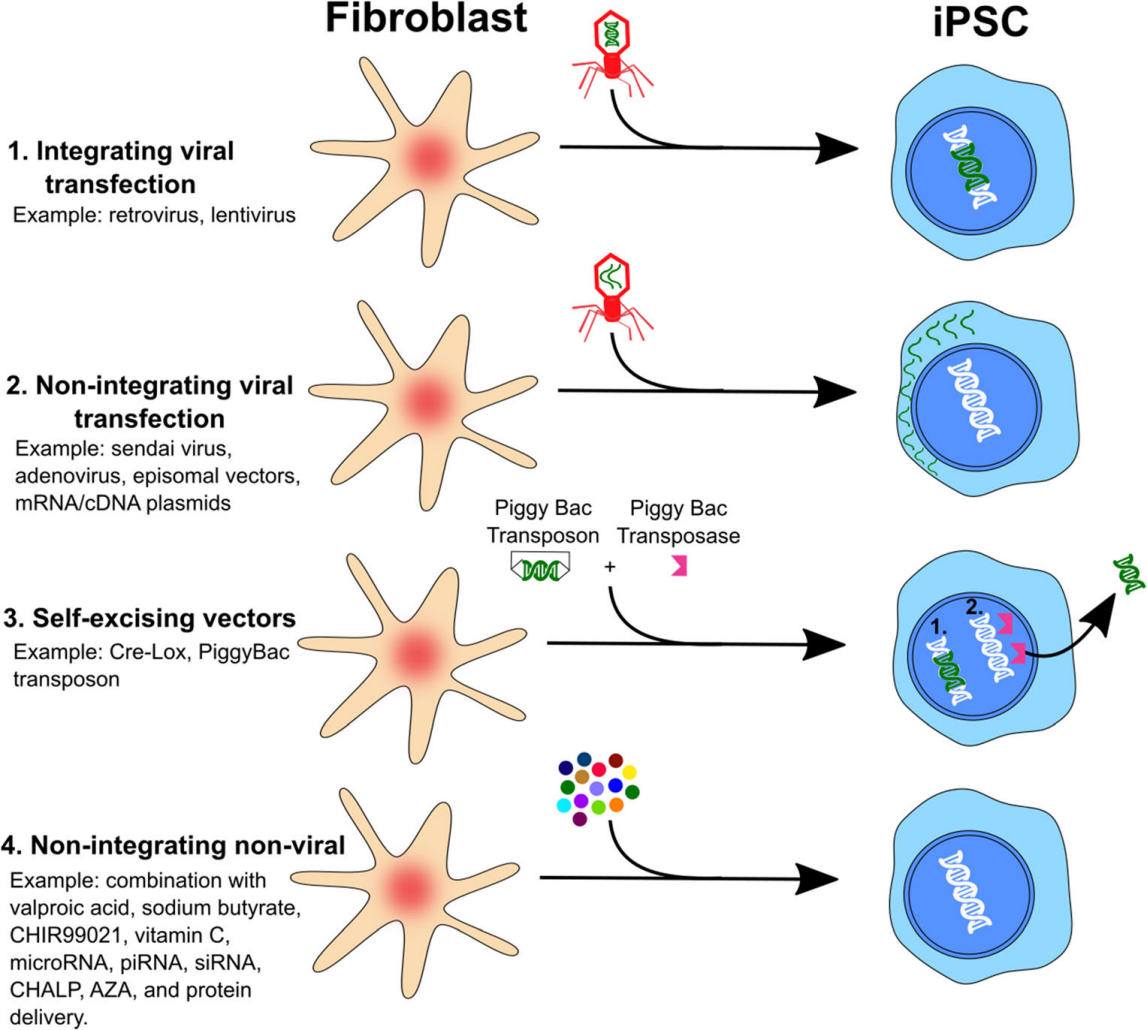
The four key methods for delivering reprogramming factors. Integrating viral systems were the first to be used to deliver transcription factors to generate stem cells, but they have the disadvantage of incorporating their genetic material and contributing to teratoma formation. By avoiding integration, novel methods (non-integrating vectors, self-excising vectors, and non-integrating non-viral vectors) represent iterative improvements upon this initial methodology. Such approaches provide significant advances in the safety and efficacy of iPSCs, which may then be applied for downstream scientific and clinical applications.

In 2006, Yamanaka and colleagues made the groundbreaking discovery that only four of the twenty-four previously used pluripotency transcription factors are necessary to reprogram mature mouse fibroblasts into an embryonic stem cell-like state, creating iPSCs (Fig. 1 and 2). These four so-called Yamanaka factors are *Oct4*, *Sox2*, *Klf4*, and *c-Myc* (abbreviated in a group as OSKM). Several years later, Yamanaka’s OSKM formula was used to generate iPSCs from human fibroblasts as well [5,74] [75],. These factors show a remarkable ability to induce pluripotency, enabling cells to develop into any of 220 cell types, at least in theory, by way of reversible epigenetic changes. Recently, Kilens and colleagues introduced a protocol that enables parallel derivation of isogenic primed and naïve human iPSCs [76]. They showed that naïve human iPSCs can be generated directly from somatic cells with OKMS overexpression and defined culture media, in a protocol with a shorter tissue culture time and more extended passages compared to previously published strategies that require priming of PSCs prior to their conversion into naive PSCs [77] [78,79],.

*Oct4* has been recognized as the most important PSC reprogramming factor, with *Nanog* and *Lin28* being effective substitutes for *Klf4* and *c-Myc*. Notably, the so-called *Oct4* complex consists of *Oct4* protein in physical association with the reprogramming factor protein products *Sox2*, *Nanog*, and *Esrrb* [62] [80],. A year after the publication of Yamanaka’s OSKM factor publication, Yu and colleagues described a modified four-factor induction protocol employing *Oct4*, *Sox2*, *Nanog*, and *Lin28*, which exhibit reprogramming with an efficiency similar to that obtained with the Yamanaka factors [81]. Additionally, due to concerns regarding the possible tumorigenic risk associated with using the proto-oncogenes *Klf4* and *c-Myc as well as* an interest in minimizing the number of factors applied, Feng and colleagues developed a three-factor method, which includes the orphan nuclear receptor gene *Esrrb* together with *Oct4* and *Sox2*; Feng’s three-factor method was shown to differentiate mouse embryonic fibroblasts (MEFs) into iPSCs with better proficiency than was obtained with the Yamanaka factors [82]. The factor *c-Myc* was shown to be dispensable for direct reprogramming of mouse fibroblasts the year prior to the introduction of Feng’s three-factor method [83]. Subsequently, the number of factors required for reprogramming has been reduced to two, including various combinations of *Oct3/4*, *Sox2*, *Klf4*, and *c-Myc* [84,85], and then reduced to *Oct3/4* alone [86–88].

The use of different transcription factors for reprogramming seems to have differing efficiency for producing specific subtypes of cells in various stages. For example, the OSKM protocol can dedifferentiate early-stage non-terminally differentiated murine B cells into a pluripotent state. Reprogramming of mature late-stage B cells, however, requires supplementary transcriptional factors, such as ectopically expressed CCAAT/enhancer-binding-protein-alpha (a myeloid transcription factor) or specific knockdown of the B cell transcription factor *PAX5* [89].

In early studies, various viral vectors, including retroviruses and lentiviruses, were used for the delivery and transduction of reprogramming factors [4] with a progressive increase in the efficiency of reprogramming [90]. Unfortunately, viral integration of transcription factor genes has the potential to produce consequential genomic alterations, including oncogenic changes in *Klf4* and *c-Myc*, which makes such protocols not amenable to clinical application [90].

The successful clinical applications of iPSCs will require overcoming serious downsides, such as incomplete reprogramming and genomic integration induced genomic alterations [91]. In recent years, iPSC techniques for removing viral vectors with non-integrating reprogramming and maximizing reprogramming efficiency have shown promise. This progress includes the recognition that various molecules, such as constructed non-viral vectors, genetic factors, signaling molecules, small bioactive molecules, microRNAs, and chemicals (described in the following section), can modulate reprogramming efficiency [82].

## Construction of novel vectors with reprogramming factors

A critical step for advancing iPSC technology is the establishment of non-viral delivery systems for introducing reprogramming factors into somatic cells. Combined with a piggyBac transposon – a single and non-viral vector plasmid comprised of a removable (eliminated from the genome by Cre) reprogramming cassette of *c-Myc*, *Klf4, Oct4, and Sox2 with the self-biomarker mOrange – has been used to reprogram somatic fibroblasts into iPSCs* [92]*. Other features of the piggyBac system have been developed that are tremendously valuable for genome-wide screening of new reprogramming factors, including piggyBac transposase-mediated excision* [93]*, high transposition activity, precise excision, and good genomic coverage* [94]*. In addition, two expression plasmids - one with Oct3/4, Sox2, and Klf4 complementary DNAs and the other with c-Myc complementary DNA - were introduced into MEFs giving rise to iPSCs without evidence of plasmid genomic integration* [95]*.*

In 2015, Schlaeger and colleagues [96] reported a systematic comparison of the three most prominent non-integrating reprogramming methods available for generating hiPSCs: Sendai-viral (SeV) reprogramming, Episomal (Epi) reprogramming, and mRNA transfection. In the SeV reprogramming system [97], SeV particles are employed to transduce target cells with replication-competent RNA molecules encoding the original OSKM set of reprogramming factors (e.g. the Cytotune kit from Life Technologies, now incorporated with Thermo Fisher Scientific Inc.). In the Epi reprogramming system [98], extended reprogramming factor expression is accomplished by Epstein-Barr virus-derived sequences enabling episomal plasmid DNA replication in dividing cells. Human Epi reprogramming was first developed in the Thomson laboratory [99], and an additional competent Epi technique was applied by Schlaeger with Oct4, Sox2, Klf4, Lmyc, and Lin28A combined with knock-down of P53 [98]. In the mRNA reprogramming system [100], cells are transfected with in vitro-transcribed mRNAs encoding the OSKM genes plus Lin28A and green fluorescent protein-encoding mRNAs. B*ecause mRNAs have a very short half-life with transfections lasting some 1-3 hours, hiPSC reprogramming requires long daily transfection procedures* [96]*. Although all three methods produced high-quality hiPSCs, substantial variance is observed with respect to aneuploidy rate, reprogramming efficiency, reliability, and workload. Reprogramming efficiency and safety for clinical translation remain challenges for these techniques.*

Relative to the other systems, SeV reprogramming is highly effective, with a lower workload and no nonappearance of viral sequences in most lines at higher passages. Meanwhile, compared to SeV reprogramming, Epi reprogramming has the advantages of a higher consistency in hiPSC generation from fibroblasts or blood samples [101] and more rapid reprogramming agent elimination. Several groups have employed small molecules [102] or used additional or modified reprogramming factors, such as BCL-XL [103] or OCT4-VP16 [104], to further boost Epi reprogramming efficiency. Schlaeger’s group in particular demonstrated significantly more effective hiPSC colony production with lentiviral (100% success rate), Epi (93%), and SeV (94%) methods compared to mRNA systems (27%, all *p* < 0.001, Fisher’s exact test).

Regarding safety for clinical translation, Schlaeger’s team suggested that Epi reprogramming was particularly well-suited for clinical translation due to it being integration-free, reliable with patient fibroblasts and blood cells, and having a very simple reagent requirement, namely plasmid DNA, which can be produced readily with Current Good Manufacturing Practice (cGMP) [96]. It has been a challenge to obtain sufficient cGMP levels under general laboratory conditions employing the same plasmids reported in the review (plasmids #27077, #27078, and #27080 from Addgene, Watertown, MA). Though the Schlaeger team has reported some data demonstrating a low-risk level [96], Epi reprogramming remains challenging. This is because of the altered genetic integrity of the resulting hiPSC lines due to the short hairpin RNA (shRNA) cassette of tumor protein p53 (TP53) after cell/tissue bioengineering. In their report, PCR data revealed that O4-shP53 plasmid sequences were reserved in 13/14 higher-passage DNA^high^ lines. The TP53 gene is the most commonly mutated gene (>50%) in human cancer, and the TP53 gene plays a vital role in averting cancer development [105]. Therefore, TP53 is categorized as a tumor suppressor gene, but its shRNA in hiPSCs functions as a silencer of TP53 expression during Epi reprogramming. Additionally, p53 plays a significant role in the maintenance of stem cells during development and as a differentiation regulator [106,107]. Indeed, TP53 and its shRNA has been shown to be extremely effective for enhancing cell reprogramming (~100 fold). This being said, it is not well suited for iPSC applications since TP53, and its shRNA in particular, may insert into iPSCs genomes, which may escape apoptosis and cause teratoma formation [108] [109],.

Notably, the major Epi reprogramming reagents provided by Thermo Fisher Scientific Inc. and Stemgent have been upgraded: CTS™ CytoTune™-iPS 2.1 Sendai Reprogramming Kit (ID: A34546) and StemRNA™-NM Reprogramming Kit (ID: 00-0076). Both kits are manufactured according to cGMP principles to enable a seamless transition to the clinic, though the latter’s efficiency requires further improvement. Research groups interested in reprogramming kits must weigh various factors when selecting an appropriate kit. For basic research, and to greatly improve Epi reprogramming efficiency, Addgene plasmids (#27077, #27078, and #27080) may be used together with additional small molecules (reviewed in the following chapter, e.g. cocktail with MEK inhibitor PD0325901, GSK3β inhibitor CHIR99021, TGF-β/Activin/Nodal receptor inhibitor A-83-01, ROCK inhibitor HA-100, and human leukemia inhibitory factor [102]); or other reprogramming factors (such as synthetic factors by fusing the VP16 transactivation domain to Oct4, Nanog, and Sox2, respectively [104]). For translational research, it is prudent to purchase the relatively inexpensive CytoTune iPS 2.0 Sendai Reprogramming Kit (Thermo Fisher Scientific Inc., ID: A16517) because it allows an easy transition to the upgraded 2.1 version for clinical applications. For clinical application, the CTS™ CytoTune™-iPS 2.1 Sendai Reprogramming Kit (ID: A34546) may be used. Although it has a lower efficiency than the Sendai kits, the StemRNA™-NM Reprogramming Kit (Stemgent, ID: 00-0076) is an appropriate option for basic research involving stem cells and specific mRNAs of interest. Notably, ReproRNA™-OKSGM Kit (Catalog #05930) is a newly launched kit by STEMCELL Technologies. It is described as a non-integrating, self-replicating RNA reprogramming vector for generating iPS cells. This single-stranded RNA replicon vector contains five reprogramming factors: *Oct4, Klf-4, Sox2, c-Myc,* and *Glis1*. Although official research reports in NCBI have not yet been published, the company claims several advantages with this kit: a non-viral, non-integrating vector system; a self-replicating vector requiring only a single transfection; the vector contains all reprogramming factors; and comparable fibroblast reprogramming efficiency to Sendai virus.

## Promoting iPSC pluripotency with molecules and genetic signaling

The combination of transcription factor-induced reprogramming with small-molecule modulation of cell signaling is a promising strategy for promoting iPSC pluripotency. Chemicals and small molecules that target signaling pathways related to cell fate, state, and function can be substituted for traditional reprogramming factors OSKM or can be used to enhance somatic cell reprogramming efficiency [110]. Hou et al. in 2013 [111] revealed the first successful reprogramming of mouse cells into iPSCs by a novel cocktail with seven small molecules (VPA, CHIR99021, E616452, Tranylcypromine, Forskolin, 3-deazaneplanocin A, and TTNPB. Furthermore, Zhao et al. in 2015 [112] promoted a 1000-fold greater efficiency by adding four small molecules (AM580, EPZ004777, SGC0946, and 5-aza-2-deoxycitidine). These mechanistic alternations of cell fate may be associated with metabolic switching from oxidative phosphorylation to glycolysis for the critical step of iPSCs reprogramming as well as small molecules substituting for Oct4 in human cell reprogramming [113]. Important details and chemical methods for generating iPSCs, neurons, cardiomyocytes, hepatocytes, and pancreatic β cells can be found in Ma’s article (2017) [113] for readers to study in greater detail.

Reprogramming can also be enhanced by induction of DNA demethylation [91]. The peptidylprolyl isomerase PIN1 regulates the induction and maintenance of pluripotency via its modulation of phosphorylation signaling [114]. The competent piggyBac transposon-based approach can produce integration-free iPSCs while satisfying the pluripotency criteria, namely pluripotency gene expression, teratoma formation in immunodeficient host mice, and contribution to chimeras [115]. Thus, teratoma formation confirms iPSC pluripotency and developmental potential, suggesting that the cells are able to produce a desired cell type [116].

The Wnt signaling pathway can also be harnessed to generate iPSCs from mouse fibroblasts. The genomic integration of the retroviruses, particularly with the gene c-Myc, increases the risk of tumorigenesis [117], and thus scientists are researching substances to replace c-Myc. The soluble small molecule Wnt modulates the Wnt signaling pathway, promoting up to a 20-fold increase in efficiency of the *c-Myc* retrovirus containing the OSKM factors [118]. Pharmacological activation of Wnt signaling with a glycogen synthase kinase 3 (GSK-3) inhibitor has been shown to favor maintenance of pluripotency in human and mouse ESCs [119], and Wnt/β-catenin signaling has been shown to regulate stem cell self-renewal and differentiation in dual dosage-dependent functions [120]. Additionally, the Wnt signaling pathway effector protein TCF3 - which colocalizes with the ESC core transcription factors *Oct4, Sox2*, and *Nanog* - has been shown to modulate the equilibrium between ESC pluripotency and differentiation [121]. RA can inhibit the canonical Wnt pathway and positively modulate Akt/mTOR signaling. Thus, two antagonistic effects of retinoic acid are present in hiPSCs: the resistance to the differentiation of hiPSCs as well as the improvement of the pluripotency state [122].

Signaling pathways mediating induction of a neuronal fate in ESCs can be controlled by bone morphogenetic protein (BMP), fibroblast growth factor (FGF), and Wnt signaling [123], with the specific neuron fate being determined by exogenous patterning signals, such as Wnt, BMP, Sonic hedgehog, FGF, and retinoic acid [122]. In response to these signals, ESCs can differentiate into a variety of neural cell types depending upon their position along the anterior-posterior and dorsal-ventral axes of the body or spinal cord [123].

Signaling pathways can alter PSC states profoundly [124]. Promoting a self-renewing state in mouse ESCs is subject to leukemia inhibitory factor (LIF) and BMP pathway signaling [125]. Self-renewal of hESCs and mouse epiblast-derived stem cells requires transforming growth factor (TGF)-β/activin/nodal signaling [126] and rat iPSCs and human iPSCs can be maintained with LIF in the presence of a TGF-β pathway inhibitor to prevent stem cell differentiation [127].

Extracellular signal-regulated kinase (ERK)/mitogen-activated protein kinase (MAPK) signaling is important for cell cycle progression, proliferation, and differentiation, and also contributes to carcinogenesis. ERK interventions have had seemingly paradoxical effects on stem cells. That is, the activation of ERK signaling has been shown to support maintenance of mouse ESC pluripotency; conversely the inhibition of MEK/ERK signaling with a MEK (MAPK/ERK kinase) inhibitor has also been shown to support self-renewal and pluripotency of mouse ESCs [128]. Additionally, mouse ESCs have been shown to be affected strongly by both MEK and GSK3 signaling [129] and simultaneous inhibition of the MEK and GSK pathways can obviate LIF and BMP requirements in PSC induction. ERK signaling has been shown to activate a shift in pluripotent ESCs from a self-renewal state to a lineage obligated state [130]. Consequently, by hindering lineage fate determination induced by the ERK signaling pathway, ESCs can be maintained in a self-renewing state [131]. The complex, and sometimes seemingly contradictory effects of ERK/MAPK interventions, could indicate a dual role of ERK/MAPK wherein, on one hand, a minimum threshold level may be required for stem cell proliferation, cell cycle progression, suppression of apoptosis, telomere length maintenance, and genomic stability. On the other hand, ERK/MAPK may repress self-renewal of mouse ESCs through downregulation of pluripotency factors and activation of developmental genes [128].

Both hESCs and mouse epiblast-derived stem cells require FGF (Yu and Thomson, 2008). Whereas hESCs require FGF2 for the preservation of an undifferentiated state [132], rat and human iPSCs can proliferate long-term without exogenous FGF2 [127]. In a model of iPSC induction involving oxygen concentration manipulation, FGF2 supplementation was shown to modulate expression of some pluripotency-related genes (e.g. *Rex1*, *Lin28*, *Oct4*, *Sox2*, and *Nanog*) at the transcriptional, translational, and cellular localization level [133]. However, this short-term induction may be insufficient for achieving true pluripotency.

Stem cells can be reprogrammed with various cocktails of small molecules such as the histone deacetylase inhibitor valproic acid [134,135], vitamin C [136], sodium butyrate [135], and the GSK-3 inhibitor CHiR99021 [127] [137],, among others. Valproic acid has been shown to dedifferentiate neonatal foreskin fibroblasts when used in conjugation with only *Oct4* and *Sox2*; interestingly, valproic acid can be substituted for the proto-oncogene *c-Myc* to prevent tumor formation [134]. Adding vitamin C to a valproic acid protocol was reported to yield approximately three times more colonies than valproic acid alone [136]. This vitamin C effect may be consequent to its promotion of DNA methylation. Sodium butyrate has been shown to be particularly effective for enhancing expression of the reprogramming factors *Ssea1*, *Sox2*, and *Nanog*, compared with valproic acid, trichostatin, and 5-aza-2'-deoxycytidine (AZA) in two pre-iPSC lines [135]. CHIR99021, when administered with *Oct4* and *Klf4* expression, can induce reprogramming of MEFs. Cotreatment of CHIR99021 with parnate (an inhibitor of lysine-specific demethylase 1) enables reprogramming of human primary keratinocyte transduced with *Oct4* and *Klf4*. These findings suggest that a GSK-3 inhibitor may obviate the need for some transcription factors in both mouse and human cell reprogramming [127]. Together, the studies summarized above validate the principle that signal transduction pathways and transcription factors can be leveraged to reprogram adult, differentiated cells into a pluripotent state.

## Induction and enhancement of cell reprogramming by RNA signaling

The process of cell reprogramming involves epigenetic alterations, including histone modification, DNA methylation, and expression of non-coding RNAs – each leading to changes in gene expression and cell fate. The establishment, maintenance, and withdrawal from pluripotency requires precise synchronization of a cell’s molecular apparatus. Considerable progress has been made in decoding several features of this intricate system, particularly with respect to transcription factors and epigenetic modifiers, as described above. In addition, RNA binding proteins mediate posttranscriptional regulation of gene expression that affects the fate of PSCs [138]. Another similar direction of cell reprogramming improvement is the use of microRNAs, which play a critical role in stem cell reprogramming and maintenance [139].

Recently, a novel stem cell culture system was discovered, termed the 5iLAF culture system. It can be used to promote naïve pluripotency in diverse types of human cells from pre-implantation embryos, to primed pluripotent stem cells, to somatic cells [140–142]. Interestingly, experiments combining a human inducible reprogramming system with the 5iLAF naïve induction platform have revealed unique transcriptional and epigenetic dynamics during human fibroblast transition to naïve iPSC. Further, they revealed previously unrecognized modes of gene network activation similar to those found during embryonic development from late embryogenesis to pre-implantation [143]. This data of naïve-induction process dynamics represent the first molecular roadmap during the reprogramming of human somatic cells into a naïve pluripotent state.

Global analysis data have revealed multiple pathways that provide specific regulation of mRNA decay in iPSCs, first by increasing the stability of histone mRNAs, second by stabilizing a large set of zinc finger protein mRNAs, and third by the destabilization of 3’UTR C-rich sequence elements in iPSCs [144]. These mechanisms underscore the importance of posttranscriptional regulation in pluripotent cells. A recently discovered class of small non-coding RNAs called Piwi-interacting RNAs have been reported to play important roles in transposon silencing, transcriptional/post-transcriptional regulation, and epigenetic modification. Epigenetic regulation of gene expression, modulation of genome stability, and regulation of chromatin status by Piwi-interacting RNAs may offer a new avenue for efficient reprogramming of somatic cells to a pluripotent state [145].

The microRNA mir-302, which is highly expressed in hESCs, has also been implicated in reprogramming [146]; and the let7 family of microRNAs has been associated with *LIN28*’s down-regulation functions that promote reprogramming [147,148]. A screening study of candidate factors that might affect reprogramming efficiency revealed that p53 small interfering RNA and undifferentiated embryonic cell transcription factor 1 enhanced the efficiency of iPSC generation from human fibroblasts by up to 100-fold, even when *c-Myc* was removed from OSKM formulas [108]. Small interfering RNAs or lentiviral short hairpin RNAs against *Dnmt1* have also been shown to be sufficient to induce rapid transition of MCV8 and BIV1 cells from a partially reprogrammed state to a pluripotent state [91].

In summary, there are many promising new reprogramming techniques and direct delivery methods, including synthetic mRNAs expressing pluripotency genes. RNA modification of the expression of genes involved in reprogramming leading to the delivery of transcription factors may replace exogenous transcription factors or enhance reprogramming efficiency [33]. Compared with Yamanaka's method, the administration of synthetic mRNAs encoding OSKM can yield a 36-fold increase in reprogramming efficiency [100]. For synthetic mRNA encoding the OSKM factors, the open reading frame (ORF) of the gene of interest is flanked by a 5′ untranslated region (UTR) containing a strong Kozak translational initiation signal, and an alpha-globin 3′ UTR terminating end with an oligo(dT) sequence for addition of the polyA tail. Thus, synthetic RNA has come to be considered a safe and efficient method of transcription factor induction for iPSC generation.

## Inducting and enhancing pluripotency in iPSCs using chemicals

Recently, chemical approaches have been developed for controlling the pluripotency and differentiation of stem cells. The classical targets for these molecules are growth factor receptors or their associated downstream kinases that regulate intracellular signaling pathways during differentiation. For example, a small-molecule antagonist of cell-surface glycosaminoglycans promotes a pluripotent state in mouse ESCs, providing a powerful new alternative to previously existing techniques for controlling stem cell fate [149].

In conventional somatic cell reprogramming without the addition of chemicals, many cells are left in an intermediate partially reprogrammed state. Supplementation of culture media with chemicals was developed to improve the efficiency obtained with reprogramming genes and with induction of the reprogramming process as a whole. The strategic combination of transcription factor transduction and chemical additives may be used to produce novel pluripotent cell types. This direction is currently an exceptionally promising area of study owing to its high efficacy, complete evasion of genomic integration, and minimization of disturbing genetic patterns.

In 2011, the CHALP molecule cocktail was reported by Yu et al. to be effective in reprogramming experiments [102]. The CHALP cocktail includes six small molecules: a GSK3β inhibitor (CHIR99021), a MEK inhibitor (PD0325901), human LIF, TGF-β/activin/nodal receptor inhibitor (A-83-01), bFGF, and a ROCK inhibitor (HA-100). Recently, another cocktail protocol has been described by Di Li in 2016 that it contains cyclic pifithrin-a (a P53 inhibitor), A-83-01, CHIR99021, thiazovivin, NaB, and PD0325901—significantly improvubg the reprogramming efficiency with 170-fold increase in human urine-derived cells (hUCs) [150]. The biological effects of the two cocktail protocols are complex. Combined treatment with the MEK inhibitor PD0325901 and LIF promotes ground state pluripotency in *Oct4* and *Klf4* pre-iPSCs [124]. Notably, PD0325901 augments iPSC production from critically transduced neural progenitor cells, promoting pluripotency and the iPSC state. It also selectively binds and inhibits MEK, which may cause inhibition of phosphorylation and activation of MAPK/ERK and thus inhibits of tumor cell proliferation [102] [151] [152],., PD0325901 promotes the growth of iPSCs while inhibiting the growth of non-iPSCs [153]. A-83-01 favors reprogramming of human epidermal keratinocytes using *Oct4* and *Klf4* by inhibition of TGF-β (smad2) [102,152]. Cyclic pifithrin-α functions to suppress or silence P53, thus considerably augmenting the reprogramming proficiency of human somatic cells [154]. Thiazovivin is ROCK inhibitor, which intensely increases reprogramming efficiency in the presence of PD, Chir, A-83-01, and hLIF [102]. Sodium butyrate stimulates miR302/367 clusters, histone H3 acetylation, DNA demethylation, and the expression of endogenous pluripotency-associated genes [155] [156],. Thus, each of these chemicals promotes the generation of a pluripotent state.

The pharmacological inhibition of DNA methyltransferases with AZA [91,157] can be used to propel pre-/partial-iPSCs toward fully realized iPSCs [82]. Valproic acid (discussed above) or AZA can also increase the kinetics of reprogramming resulting in faster attainment of fully proficient iPSCs. Valproic acid also empowers effective induction of PSCs without introduction of the oncogene *c-Myc* [134]. Valproic acid is recognized for its ability to improve reprogramming efficiency by more than 100-fold, as indicated by an *Oct4-GFP* reporter [84]. Other histone deactylase inhibitors, such as trichostatin A (up to 15-fold increase in efficiency with OSKM) and suberoylanilide hydroxamic acid (∼2-fold increase in efficiency with OSKM), also augment reprogramming efficiency [84]. Another small-molecule combination, BIX-01294 (G9a histone methyltransferase inhibitor) and BayK8644 (L-type calcium channel agonist), enable reprogramming of *Oct4/Klf4*-transduced MEFs [157]. The glucocorticoid analogue dexamethasone increases the effect of AZA by 2.6-fold during induction of mouse fibroblasts to iPSCs [134].

In summary, bioactive chemicals are being used to enhance reprogramming or even to replace core reprogramming factors. These factors hold exciting potential to significantly advance the field of stem cell and regenerative medicine.

## Maintenance and modification of pluripotency and genomic stability in iPSCs

A great variety of factors, including the cell’s genetic makeup (genotypes) and external factors (environmental epigenetics), may produce previously unobtained phenotypes. Epigenetic mechanisms, including DNA methylation and histone modification, can be initiated exogenously to produce enduring variations in gene expression and thus influence phenotype [158]. These modifications may be a driver of chromosomal aberrations, mitochondrial mutations, genetic diversification, and epigenetic variance [159]. They increase biological plasticity that shapes future gene expression in response to changing environments and conditions, including disease development. Similarly, genetic and epigenetic factors can modulate differentiation tendency in PSCs. These principles apply to iPSCs that were reprogrammed from mature cells as well [159].

There may be genetic and epigenetic variations among different iPSC lines [160]. Dissimilarities may be inherited from donor somatic cells or produced during reprogramming or culturing [160]. There is evidence that epigenetic memories or incomplete reprogramming may disturb iPSC differentiation properties [161] [162],. If aspects of the genome associated with iPSC properties are affected, the functional activity of iPSC derivatives may be impaired, a mixed population of differentiated cells may be obtained, there may be residual undifferentiated cells, and there could be an increased risk of tumorigenicity [161] [162],. Thus, reprogramming strategy and culture conditions must be optimized to minimize such variations [163].

Utilization of PSCs in regenerative therapy will require pluripotency with unrestricted self-renewal but without concomitant chromosomal instability [164]. Maintenance of telomere length is crucial for unrestrained self-renewal, pluripotency, and chromosomal stability of PSCs. In addition to telomerase, which plays a key role in telomere maintenance, there are several pathways required for telomere lengthening that are linked to genetic recombination and epigenetic modifications. Telomere reconstruction is an aspect of epigenetic reprogramming that is vital to pluripotency. Understanding telomere reprogramming and maintenance in PSCs has ramifications for aging and tumorigenesis [164]. Telomeres preserve chromosome constancy and cell replicative capability. Telomere length is determined by the balance between telomere elongation and telomere reduction [165]. The reprogramming of differentiated cells induces T-circle and single-stranded C-rich telomeric DNA accumulation, which activates telomere trimming pathways that compensate for telomerase-dependent telomere elongation. Telomeres are longer in PSCs than in somatic cells, and telomere elongation through reprogramming is critical for achieving authentic pluripotency [166]. SIRT1, a member of the sirtuin family of NAD+-dependent lysine deacetylases, plays a key role in proficient telomere elongation and genomic stability of iPSCs [167], while telomerase reverse transcriptase is used in somatic cell reprogramming [168].

Experiments have demonstrated that iPSCs and hESCs exhibit similar defense mechanisms and mitochondrial regulation processes to prevent the production of DNA-damaging reactive oxygen species, which confer cells with comparable competencies to sustain genomic integrity [169]. The DNA damage response is critical for maintaining genomic integrity. PSCs derived through more effective reprogramming approaches hold additional hESC-like activated *c-Myc* signatures as well as DNA damage response signaling [170]. A faithful *c-Myc* molecular signature could serve as a biomarker of genomic integrity in hiPSCs. Cyclin-dependent kinase 1 regulates multiple events in hiPSCs ranging from mitosis regulation, G2/M checkpoint maintenance, apoptosis, maintenance of pluripotency, and genomic stability [171].

Failure to repair double-strand breaks in DNA not only compromises the capability of stem cells to self-renew and differentiate but can lead to genomic instability and eventually disease. Two properties of PSCs in the early reprogramming phase may compromise genomic stability [172]. The first property is that PSCs have a high proliferation rate and a short G1 phase in the cell-cycle [173]. The second is that PSCs profoundly depend on anaerobic glycolysis rather than oxidative phosphorylation [174]. Furthermore, during the cellular reprogramming process, reduced mitochondria activity is insufficient to remove reactive oxygen species (ROS) generated by increased cell proliferation, thus resulting in oxidative stress. Consequently, challenges exist during the proliferation and differentiation phases as well.

Relative to somatic cells, ESCs have distinct mechanisms for defending against double-strand breaks and oxidative stress [175]. ESCs represent the point of origin of all cells to develop organism and thus, must protect their genomes from both endogenous and exogenous genotoxic stress. A vigorous DNA repair response to endogenous and exogenous stress is vital to sustain the genomic integrity of ESCs and guarantee accurate differentiation program. However, during reprogramming, iPSCs seem to be susceptible to genotoxic stress. ESCs have specialized mitochondrial features, but fewer and poorly defined mitochondria, when compared with mature cells [175]. Thus, ESCs display hypersensitivity to DNA damage [176]. This being said, ESCs can control intracellular ROS concentrations [177] and they have exclusive mechanisms to uphold a highly error-free form of DNA double-strand breaks repair. However, DNA double-strand breaks response may not be completely processed in all iPSCs throughout reprogramming. DNA single-strand breaks resulting from ROS and other agents can lead to double-strand breaks during replication. Further, DNA double-strand breaks-associated with the DNA damage response may be associated with ataxia telangiectasia and Rad3-related protein (ATR) and other gene ataxia telangiectasia mutated (ATM)-independent mechanisms. Such mechanisms are imperative in ESCs to preserve high genetic integrity under genotoxic stress [178]. Thus, adequate responses to stress and harm are critical for the maintenance of stem-cell self-renewal, differentiation capacity and genomic stability for stem cells. However, this presents a unique challenge for iPSCs.

Genomic instability of iPSC can occur at any processing stage, causing mutations of the final cell products, which may have implications for clinical transplantation. Recently, in 2017, Yoshihara et al., summarized the genomic instability of iPSCs, thus challenging their potential clinical applications [179]. They found at least three origins for such genomic instability: (a) pre-existing variation, where changes in allele frequencies (~50%) in parental somatic cells may be caused by a cloning step during iPSC generation; (b) reprogramming-induced mutations, whose allele frequencies are 25% and 12.5% after first- or second-cell division, respectively; and (c) passage-induced mutations arise during prolonged culture at low allele frequencies. Thus, genomic instability can pose significant challenges for iPSC integrity.

In 2019, Doss and Sachinidis first proposed the ten minimum quality criteria required for clinical-grade iPSCs and their differentiated products [180]. These include: (1) sterility, cGMP, and freedom from mycoplasma and other endotoxins; (2) expression of pluripotency-associated marks such as *Nanog, Oct4, SSEA-3, SSEA-4, TRA-1-60, TRA-1-81*, and Sox cannot be detected; (3) expression of differentiation markers must be unique to the therapeutic cellular product; (4) normal karyotype and absence of chromosomal aberrations must be present; (5) the absence of undifferentiated iPSC in the final cellular drug product and freedom from tumorigenicity as confirmed by *in vivo* teratoma assay and whole-genome and exome sequencing, as well as flow cytometry; (6) 100 % purity of the therapeutic cellular product without any contaminating foreign lineage cell types; (7) *in vivo* data on cell engraftment showing durability and functional improvement in preclinical models; (8) no residual reprogramming transgenes and vectors can be detected by whole-genome or exome sequencing; (9) genotyping in cases of autologous iPSCs can be demonstrated by short tandem repeat analysis; and (10) viability must be present in the context of clinical-grade stem cell products.

Genetic factors may modulate iPSC fate, including whether a desired normal cell phenotype (e.g. neuron or cardiomyocyte) or undesired cell phenotype, such as a non-specific or cancerous type of cell, is obtained. Such influences can be biologically significant in the context of clinical translation of iPSC and and iPSC-derived cell protocols. If transplanted cells develop into undesired cells, such as non-specific normal cells or cancer cells, or migrate to unintended places, there could be serious health consequences. Thus, the maintenance of pluripotency and genomic stability in iPSCs is critical for the safety of downstream clinical applications.

Employing iPSCs in research and clinical applications will require the ability to modify pluripotency and genomic stability. In addition to reprogramming with small molecules, microRNAs, and reprogramming factors, there has been a recent interest in modifying the genomic stability of stem cells to create disease models by combining two advanced technologies: hiPSC generation and CRISPR (clustered regularly interspaced short palindromic repeats)/CRISPR-associated gene (Cas) technologies [181]. The state-of-the-art CRISPR/Cas9 genome editing method has revolutionized biomedical research, stem cell biology, and human genetics. It enables gene expression to be modified through CRISPR interference or CRISPR activation by reversibly directing a target endogenous promoter. It provides a means of introducing reporter genes or achieving ectopic expression. With CRISPR/Cas protocols, genetic information can be deleted or inverted by single base-pair changes that introduce a mutation or polymorphism, or even repair a disease-relevant mutation. Parallel differentiation of CRISPR/Cas genetically engineered hiPSCs and wild-type cells (for comparison) provides a basis for phenotypic analysis of disease-specific cellular pathologies. This approach can reduce animal model usage and save time and money, while also improving quality control with respect to reproducibility and stability. A series of CRISPR-Cas9 system experiments demonstrated the role of **the** jumonji and AT-rich interaction domain-containing 2 genes **in self-renewal in hESCs** [182]. The CRISPR/Cas9 system was shown to enable scarless introduction or correction of disease-associated variants in hPSCs, thereby combining genome editing and stem cell technologies to construct genotypic “disease-in-a-dish” models [183,184]. Such genome-editing approaches are referred to as scarless because they are applied to genotype-specific disease models using only intended DNA base-pair edits without extra-genomic modification. The genomic stability of stem cells can also be modified with CRISPR/Cas9 technologies to generate new disease models as novel areas of research [183,184]. These methods can be used to establish precision disease models for drug screening, making them highly promising for regenerative medicine.

## Feeder-free and xeno-free culture environments

For clinical translation, culturing iPSCs in feeder-free conditions is of utmost importance [185] (see Table 2). Thompson’s gold-standard self-renewal culture technique calls for placing iPSCs (mouse or human) on a monolayer of feeder-cells, such as primary mitotically inactivated MEFs [3]. Long-term maintenance of hPSC cultures was accomplished using scalable, stable, and cost-effective poly(acrylamide-co-propargyl acrylamide)-coated polystyrene flasks with coupled cRGDfK coating (with modifying two-polymer brush coating [poly(acrylamide-co-acrylic acid) and poly(acrylamide-co-propargyl acrylamide)] [186]. Although Matrigel is a beneficial substitute material for culturing hPSCs [187] [188],, it is derived from a mouse source [189]. Other matrices, such as CellStart [190,191], recombinant proteins [192] [193,194],, and synthetic polymers [195] [196], that do not involve animal-derived products are preferred for use when culturing iPSCs.

**Table 2:** Critical comparisons of cell culture, medium, and material for iPSCs growing environment

	**Feeder-free cell culture**	**Xeno-free medium**	**Biomaterials**
Definition	Plates, wells, and culture are cell-free with the exception of the desired cell type.	Serum-free culture	Material, mechanical, or biological technologies for coating plates/wells to promote growth, maintenance, or differentiation [219]
Key Substances	Thompson’s inactivated MEFs (gold standard) [3]	No animal-derived elements, but contain minimal growth factors [199]	Biodegradable polyester-based materials [210] [211], and nano-/microparticles formulated from poly-lactic-co-glycolic acid with FDA approval [212] [213],
Advantages	MEFs secrete vital growth factors including FGF, TGF-β, cytokines, and extracellular matrix (ECM) proteins (e.g. activin A, laminin-511, and vitronectin) [320] [321],	To avoid contaminating cultures with unknown proteins or zoonotic viruses; to manage appropriate growth factors for forced differentiation or therapy [197]	May improve safety, efficiency, and scalability limitations of conventional iPSC derivation by controlling iPSC behavior *in vitro* and *in vivo* [261]
Other requirements	Need growth-suppressive (mitotically inactivated) treatments such as mitomycin, γ-irradiation, electric pulses, or chemical fixation [322]	Insulin, transferrin, and selenium [199]	Low toxicity and biodegradability
Risks	May contaminate cultures with unknown proteins or zoonotic viruses [322,323]; MEF expression and secretion of growth factors are inconsistent; Anti-proliferation treatments may lead to apoptosis [321,324,325]	Potentially disrupt differentiation or therapeutic capacity [197]	To identify and characterize biomaterial properties that are compatible, promotable, non-toxic, and degradable for the transplant
Solutions	Synthetic culture surfaces such as recombinant human vitronectin-N–coated dishes or biomaterial coating [326]	Xeno-free nutrition supplements such as ThermoFisher Scientific N2 (A1370701) and B27 (A3353501)	The development of novel biomaterials

iPSC culture media should also be well defined, xeno-free, and serum-free, all of which may improve stem cell differentiation capacity [197] (see Table 2). Numerous studies have shown successful stem cell culturing under xeno-free conditions. For instance, use of a growth factor-free, chemically-defined medium was reported to be important for the induction of rostral hypothalamic-like progenitor cells from neuroectoderm-derived mouse ESCs [198]. Meanwhile, PI3K/AKT- and Ras/MAPK-dependent signaling pathways were reported to sustain pluripotency and viability in hiPSCs cultured on Laminin-511 in serum-free medium [199]. Dissociation with serum-free EDTA/PBS has also been reported to produce small cell aggregates with high survival efficiency and cryopreservation in a time-efficient manner [200].

Scalable microcarrier-based manufacturing using xeno-free media and bioreactors can also be used to generate mesenchymal stromal cells (MSCs) [201]. The inability of two-dimensional planar technology to produce cells of adequate quality and quantity necessitated a shift to serum-free microcarrier cultures, which require optimization of several factors including tissue source, medium formulation, microcarrier type and matrix, and agitation regime. Optimizing these parameters is critical for successful bioreactor-scale production of MSCs for cell therapy [202].

Clinical grade MSC production adhering to cGMP and quality control standards are needed to ensure the delivery of cell therapies that are safe, reproducible, and efficient. Human platelet lysate has been suggested to be the gold standard for human cell propagation, replacing animal serums in a growing spectrum of applications because it has abundant growth factors and cytokines in platelet granules. These can be released naturally by thrombin activation or artificially by frozen/thawed platelet lysis, sonication, or chemical treatment [203,204]. However, human platelet lysate may not be practical for daily laboratory work. There is significant concern over the risk of xenopathogen contamination, which would make hESCs unsafe for regenerative medicine [205]. Xeno-free products are being developed in an effort to obviate this risk [206].

There is ongoing debate regarding whether the surrounding space in stem cell cultures should be static (traditional method) or moving/stirred (novel method). ESCs can be cultivated in stirred microcarrier cultures, which represent a robust scalable pluripotent cell expansion system [207]. Such moving cultures can produce high concentrations of murine ESCs, 10-fold greater per medium volume, and 5-fold greater concentration per surface area, compared to static cultures. Furthermore, xeno-free microcarrier bioreactors have been engineered for stirred-suspension hPSC cultivation [208]. Microcarrier stirred-suspension bioreactors represent an attractive model for scalable hPSC expansion and differentiation. Although the precise mechanisms underlying the benefits of stirred stem cell culturing are not known, it is reasonable to suppose that cells have a distributed supply of nutrition owing to the circulation of medium. Additionally, the physical stimulation may favor growth. A fundamental base for three-dimensional (3D) cell culturing (discussed in a later section) has been developed based on these ideas [207,208]. Ongoing research is testing the proposition that moving cultures should replace static cultures.

## Biomaterials

Biomaterials—that is, materials that intermingle with biological systems [209] can provide an effective experimental strategy for iPSC research and application (see Table 2). Biomaterial strategies may provide novel approaches to minimizing risks related to residual undifferentiated iPSCs or malignant transformation after transplantation [210] [211] [212] [213],., ,Additionally, these platforms may improve reprogramming efficiency and factor delivery [214] [215],. In addition to genetic factors, signaling molecules, small molecules, microRNAs, and chemicals as discussed above, biomaterials offer a promising approach to increasing reprogramming efficiency and scalability. Upon reprogramming, iPSC growth and differentiation can be improved by using a stem cell niche, that is, an environment that mimics the natural microenvironment of stem cells and thereby modulates stem cell phenotype development, proliferation, and differentiation [216]. A stem cell niche may include defining ECM structures, 3D architecture, chemical and mechanical signals, and cell-to-cell interactions [217,218]. Additionally, biomaterials can govern the kinetics of reprogramming factors via nanoparticle- and microparticle-based systems [219–221], and they can regulate stem cell fate and function [219] [221],. Biomaterials may also be employed to facilitate iPSC transplantation [222] [223] [224],.,

Poly(N-isopropylacrylamide)-co-poly(ethylene glycol) hydrogel has shown particularly good efficacy in encouraging long-term iPSC expansion with a high growth rate, adequate purity, and fidelity of pluripotency in a fully defined and scalable 3D culture system for human PSC expansion and differentiation [225]. In addition, this hydrogel was shown to support differentiation into cells from all three germ layers as well as teratoma formation following long-term expansion *in vitro* and *in vivo*, respectively [226]. The robustness of this system was validated in multiple hESC lines.

Modifiability and reasonable scalability can be achieved with the various biomaterials that are currently available, overcoming to some extent the limitations of traditional substrates without bioactive materials. Of all investigated biomaterials, the synthetic polymer-based expansion platform have proven to be extremely valuable for establishing stem cell culture scalability [227] [228],. Furthermore, combining synthetic substrates with biomolecules, such as growth factors, may encourage iPSC development by enhancing material interface-mediated signaling, which is critical for stem cell self-renewal. For instance, LIF on polyester fiber substrates [229] or surfaces pre-engineered with a growth factor linker [230] presented substantial benefit for ESC expansion in the presence of a small number of growth factors. Recent data have suggested that LIF plays an important role in neuronal development. For example, LIF-dependent induced primitive neural stem cells can be expanded to >100 passages, and with such long-term culturing these cells can differentiate into motor neurons, dopaminergic neurons, astrocytes, and oligodendrocytes, indicating a high level of plasticity [231]. Alternatively, iPSC self-renewal can be promoted on standard tissue culture grade polystyrene substrates by attaching substrate-induced protein adsorption/cell adhesion without supplementary chemical modifications [232].

Furthermore, mechanical biomaterials, such as matrices, can regulate the differentiation of PSCs. Artificial bioengineered peptides are alternatives to scaffolding ECM components. A genetically engineered ECM protein product, consisting of integrins and cadherins, can provide efficient coating on hydrophobic tissue culture plates, providing a novel approach for iPSC expansion *in vitro* [233]. The mechanical properties of the underlying matrix promote robust differentiation of hESCs into neurons without neurogenic factors [234]. Moreover, engineered natural and synthetic surfaces with topographical features can be used to augment iPSC adhesion, induce neuronal differentiation, and direct axon growth [235] [236,237],. Cell-to-cell interactions and cell-to-ECM interactions, such as through laminin or collagen, have been shown to influence neuronal differentiation of neural stem cells [238]. Conversely, recombinant human laminin 521 stabilizes hESC pluripotency [239].

To improve the effectiveness of neural-inductive moieties and promote iPSC neurogenesis, biomaterials can be chemically enhanced. For example, synthetic neurotransmitter analogs have been added to promote neuronal-fate differentiation of iPSCs [240] [241],. Promising practical advances include biomaterials that can control the presentation of neuron-inductive growth factors in a sustained fashion. For instance, since a mussel adhesive protein at mussel adhesive pads can promote attachment to virtually any type of organic or inorganic material, the mussel biomimetic approach has been applied to surface modification to yield efficient human neural stem cell differentiation and proliferation [242]. In addition, hybrid-polyester scaffolds with heparinized surfaces support neuronal differentiation of iPSCs [243].

To accomplish large-scale iPSC expansion (billions of cells) for downstream applications, including clinical applications, the aforementioned synthetic substrates could be engineered into microcarrier/suspension bioreactor systems [244]. Moreover, synthetic materials can be customized easily into high-throughput platforms [195] [245],. The use of high-throughput biomaterials/ECM screening technologies [195,246] with computational modeling [247] can empower current and future research.

## Application of 3D cell technology

The development of 3D cell technologies for iPSC protocols has emerged as an exciting new field. For example, 3D ESC spheroids have been shown to produce efficient mesoderm induction in the presence of approximately 1/12^th^ the amount of total growth factors in traditional iPSC cultures [248]. Compared to 2D monolayers, 3D culture systems offer the benefits of native cell-cell and cell-matrix interactions that improve the efficiency of spatial-temporal signals [249] [250], essential for cell proliferation and functioning [251]. Additionally, 3D culture platforms augment the space available for cell proliferating, thus enabling a scaling up of iPSC expansion without triggering the formation of unfavorable clusters and yielding higher cell densities and larger spheroids than 2D systems [252]. Expansion of iPSCs has been proven to be highly efficient in 3D scalable, synthetic platforms [225].

The approaches reviewed above may improve the utility and effectiveness of iPSC growth and directed differentiation into desired functional cell subtypes. Biocompatible chemically demarcated synthetic controllable substrates can serve as next-generation substrates for large-scale iPSC production with cGMP compliance, which is well-suited for real-world clinical applications [253] [254],. Synthetic, scalable, chemical 3D matrices are a highly promising platform for both basic scientific investigation and clinical iPSC applications.

## Use of 3D bioprinting stem cell technology

In recent years, 3D bioprinting has attracted attention for its promise in the manufacture of iPSC-derived organ systems. It allows layer-by-layer prearrangement of biomaterials, biochemicals, and living cells with accurate spatial control [255] [256],, thereby mimicking the systemic complexities of physiological or pathological conditions [257] [258],. This technology is based on living cell cultures, biocompatible materials, and supporting instruments, including computer-controlled high-throughput technology. Efforts are well underway to produce 3D functional tissues and organs that can be used for tissue modeling (“organ-on-a-chip”) applications and clinical transplantation [146] [259] [260],., Such developments offer a potential future solution to the insufficient donor tissues and organs available for liver, heart, and vascular transplantation. Organ-on-a-chip platforms are useful for toxicological and pharmacological testing new candidate drugs on target tissues. Biomaterials provide microenvironmental elements for living cells, the backbone for the printed constructs, and protect living cells during printing [261] [262],. The advantages of 3D bioprinting have been fully demonstrated in fugitive/sacrificial and cell-laden hydrogel ink materials [263] and 3D bioprinting platforms are a promising tool for generating functional tissues or organs, which may be used for therapeutic drug screening, tissue morphogenesis research, and establishing physiological stem cell niches in 3D bio-printed iPSC arrays.

## Downstream stem cell applications

The ultimate goals of stem cell technology is application in regenerative medicine, disease modeling, drug screening/discovery, and human developmental biology. Toward these aims, reprogramming technology to generate iPSCs has developed momentously in recent years. In neuroscience, this technology has the potential to treat SCI [264] [265] [266],,, brain injury [267] [268] [269],,, Alzheimer disease [270] [271],, PD [272], and amyotrophic lateral sclerosis [273]. It has many advantages, including resolving cell shortages owing to readily accessible cell types (e.g. fibroblasts from biopsied skin or urine samples), being reprogrammed in culture, and personalization for clinical use, thereby obviating or reducing the need for immunosuppressive therapy and any associated risks [274] [275],.

Another outstanding benefit of iPSCs is that some cell subtypes can be reprogrammed directly to generate *in vitro* disease models of cells compromised or destroyed by disease processes, such as in amyotrophic lateral sclerosis [273], sudden spinal cord trauma [264] [265] [266],,, and stroke [267] [268] [269],,, as well as models of cells subject to degenerative processes in Alzheimer disease [270] [271], and PD [272]. The iPSCs derived from a variety of genetic disease sources with Mendelian or complex inheritance (i.e. adenosine deaminase deficiency-related severe combined immunodeficiency, Shwachman-Bodian-Diamond syndrome, Gaucher disease type III, Duchenne muscular dystrophy, Becker muscular dystrophy, PD, Huntington disease, type 1 diabetes mellitus, Down syndrome, and the carrier state of Lesch-Nyhan syndrome) have been closely investigated [75] [272],. Such disease-phenotypic iPSC models can be used to recapitulate pathologic mechanisms and to investigate candidate drug, genetic, and cellular replacement therapies [276] [277],.

For example, iPSCs derived from a patient with amyotrophic lateral sclerosis were directed to differentiate into replacement motor neurons for cell therapy [273]. Amyotrophic lateral sclerosis (ALS) is a mostly sporadic disorder (90%), but a few cases (10%) can be autosomal dominant and rarely X-linked or recessive [278]. More than 20 mutated genes have been identified, including SOD1 [279], TDP-43 and C9ORF72 [280].

iPSCs can be differentiated from ALS somatic adult cells such as dermal fibroblasts by reprogramming factors that preserve pluripotency. Further, hiPSC differentiation into active motor neurons has been documented in vitro and in vivo [281,282]. Noticeably, in the asymptomatic SOD1^G93A^ rat model, neural progenitors (NPs) has been successfully identified following injection of hiPSCs into the ventral horns of the lumbar spinal cord [283]. In this model, the potential iPSC-derived NPs survived for 10 days after intrathecal injection, increasing survival by 23 days following systemic intravenous cell infusion , when compared to control PBS injected mice [284]. Additionally, the protective trophic factors such as GDNF, BDNF, NT-3, and TGF-α were secreted in the transplanted cells to protect resident motor neurons and reduce astrogliosis [284]. To evaluate the efficacy of transplanted stem cell engraftment, advanced microscopy techniques such as confocal and two-photon microscopy could also be helpful tools when tracking *in vivo* models [285].

Importantly, the limitations of these techniques include questions regarding administration route, optimal dose, differentiation state, neuroprotective mechanisms, as well as an appropriate time of cell injection according to disease onset [286]. Since various genes can be mutated in ALS [273], such as SOD1 [279], TDP-43, and C9ORF72 [280], each ALS model represents only a subpopulation of patients with ALS. Thus, future studies need to account for these limitations.

Animal model studies have demonstrated the beneficial effects of stem cells. For example, dopamine neurons derived from hESCs have been engrafted efficiently in animal models of PD [272]. hESC-derived retinal pigment epithelium has also been shown to improve vision in models of blindness [287], and iPSC-derived cardiomyocytes have been shown to improve cardiac function in a porcine ischemic cardiomyopathy model [288].

The ultimate goal of stem cell research is clinical application in patients. There are several ongoing stem cell clinical trials around the world, including studies targeting bone/cartilage, heart, neural, immune/autoimmune, kidney, lung, liver, gastrointestinal, and metabolic diseases [289] [290],. There is particular interest in hESC- and hiPSC-derived product studies focused on SCI, PD, macular degeneration, type 1 diabetes mellitus, and severe heart failure [289]. With respect to clinical trial phase, the largest portion, 40.6%, of 352 registered clinical trials are phase I/II with 26.0% being phase I, 22.5% phase II, 6.7% phase III, 3.8% phase II/III, and 0.3% being phase IV [290].

The first clinical trial (ID: NCT01217008) to assess the safety of hESC-derived oligodendrocyte progenitor cells (OPCs) in SCI therapy was launched by Geron Corporation in 2010 with OPC1 cells, which were the first hPSC type isolated two decades ago by Dr. James Thomson at the University of Wisconsin [3]. The results obtained by Geron were presented at the American Society for Gene and Cell Therapy meeting in 2014 [290]. They showed no serious adverse events one year after transplantation in five participants and magnetic resonance imaging revealed alleviation of spinal cord deterioration in four of the participants. Asterias Biotherapeutic (AST) continued the Geron study from March 2015 to December 2018 (SciStar clinical trial, clinical trial ID: NCT02302157, https://clinicaltrials.gov/ct2/show/NCT02302157). In preclinical safety experiments, cell culture and animal model data identified AST-OPC1 cells as an early-stage oligodendrocyte progenitor population capable of facilitating neurite outgrowth *in vitro* and myelination *in vivo*, without adverse sequelae, such as toxicities, allodynia, or tumorigenicity [291]. AST-OPC1 cells improved locomotor function (as indicated by automated TreadScan monitoring) when administered directly into the cervical spinal cord 1 week after SCI in rats; this functional improvement was accompanied by reduced parenchymal cavitation and increased myelinated axon sparing at the injury site [292]. All preclinical safety and efficacy data thus far support commencement of an AST-OPC1 Phase I clinical trial in patients with sensorimotor complete thoracic SCI.

The SCiStar phase I/IIa study exploring a novel and innovative therapeutic approach is underway at nine US hospitals with 35 enrolled participants. It is an AST-OPC1 dose escalation study in SCI officially titled, “A Phase 1/2a Dose Escalation Study of AST-OPC1 in Subjects with Subacute Cervical Spinal Cord Injury”. The initial results (available on the company website at http://asteriasbiotherapeutics.com/) have demonstrated significant efficacy of this therapy for SCI, including improvements in running speed, forelimb stride length, forelimb longitudinal deviation, and rear stride frequency. All participants will have be followed up to 14 years to enable long-term evaluation of the effectiveness and any adverse secondary effects of the therapy.

Taking a momentous step toward regenerative medicine with iPSCs, the first successful transplant of iPSC-derived retinal cells was performed in the eye of a 70-year-old woman suffering from advanced macular degeneration [293]. The patient received a transplant of ~250,000 retinal pigment epithelial cells generated from autologous iPSCs. After testing the tumorigenic potential of patient-28-RPE cells by using immunodeficient mice (nonobese diabetic/Shi-scid/IL2rγ^null^ [NOG] mice), examining whole-genome sequencing for potential mutations, and investigating the integration of plasmid DNA into the host cell genomic DNA, all results demonstrated that transplanted cells were genetically stable. Additionally, there were no serious side effects and no sign of rejection during the 1-year study period in 2016. Recently, in April 2019, Masayo Takahashi and her team updated the 4-year follow-up [294]. The function of the grafted autologous iPSC-derived retinal pigment epithelium cell sheet was evaluated by color fundus photography, spectral-domain OCT, fluorescein angiography, indocyanine green angiography, and an adaptive optics retinal camera. As a result, the cells have survived for 4 years, support and nourish photoreceptors and choroidal vessels, and display the morphologic characteristics of the retinal pigment epithelium at the transplant site. However, this study was a clinical case study rather than a real clinical trial, and thus, the safety and efficacy of this therapy need to be further investigated.

Another major application field for iPSCs is the large-scale screening of chemical libraries for systemic disease-treating drug discovery. Several iPSCs derived from patients with neurological and psychiatric conditions are currently being investigated for drug screening [295]. An iPSC model of a fully penetrant aggressive form of PD (synuclein alpha triplication, encoding α-synuclein) has been established to identify compounds that reduce α-synuclein levels [296]. Additionally, human dopaminergic neurons derived from iPSCs carrying the most common PD-causing mutation (p.G2019S) in *LRRK2* (leucine-rich repeat kinase 2) have been developed [297]. Importantly, they show increased expression of oxidative stress-response genes and α-synuclein protein. Several mitochondrial response metrics (reactive oxygen species, respiration, proton leakage, and intraneuronal mitochondrial movement) were assessed in iPSC-derived neural cells carrying *PINK1* (PTEN-induced putative kinase 1) and *LRRK2* mutations (from patients with familial PD), showing pharmacological rescue of mitochondrial deficits [298]. Such cells, originally differentiated from iPSCs that were derived from adult somatic cells, offer an attractive platform for drug and toxicity screening in preclinical trials. Added benefits of these techniques include reducing the use of animals and costs while saving time. For example, a 7-day high-throughput/high-content screening assay protocol for identifying compounds that affect hESC self-renewal and differentiation in 384-well plates has also been developed with some success [299].

Another stem cell research area is neurodevelopmental disorder modeling in hPSCs [300] [301],. In neurodevelopmental disorders, nervous system growth and development are altered *in utero* and during early postpartum life. Because hPSCs maintain the unique genetic signatures of the individual from whom they were derived, they may be able to recapitulate, to some extent, that individual's characteristic neural development, including impairments in neurogenesis and synaptogenesis that may underlie intellectual disabilities and delayed neurodevelopment [300] [301],. iPSC technology can be used to study neurogenesis [302], that is, the proliferation and differentiation of neural stem cells into glia and neurons, which are then organized into exquisitely regulated functional networks. For example, bipolar disorder is a chronic neuropsychiatric disorder characterized by pathological fluctuations in mood between mania and depression. Studies of iPSC lines from patients with bipolar disorder have revealed alterations in calcium signaling, neuronal differentiation, glial lineage specification, and some impairments associated with WNT, Hedgehog, and Nodal pathway signaling [303].

Furthermore, combining stem cell technology with genomic editing tools such as CRISPR may establish an unprecedented modeling system in mammalian neural development and brain organoid research [304]. For instance, Huntington disease, a debilitating heritable condition, occurs because CAG repeats encode for glutamine in the huntingtin gene [305], wherein more repeats are associated with greater severity and an earlier age of onset. Combining organoid and CRISPR gene editing methods may help to elucidate the mechanisms of genetic repeat diseases [304]. In late 2018, Chinese scientist He Jiankui at The Second International Summit on Human Gene Editing in Hong Kong made an announcement that his team has successfully carried out human germline gene editing by using CRISPR technologies to create gene-edited twins to “prevent” HIV infection [the website of The Second International Summit on Human Gene Editing].

Organ transplantation is considered the final therapy for organ failure, but there is an extreme shortage of organ donors, and transplantation requires donor-recipient matches. Thus, an alternative source of cells and tissues, such as iPSCs, could help to solve these challenges [306]. 3D stem cell structures built with biomaterials and bioprinting technology may enable organ reconstruction in the future [307]. Although complete, functional organs have yet to be reconstructed, parts of organs have been reconstructed, including partial livers [246] [308],, vasculature [309] [310],, bone [311] [312],, and proto-bladders [313] [314],. Organ reconstruction requires two parallel complementary stages: de-cellularization, during which the structural integrity of the extracellular matrix and vascular network of the organ must be maintained after washing out and removing waste tissues with biochemical detergents, physical forces (e.g. agitation), and re-cellularization – during which the remnant organ scaffold retaining the full network of blood vessels and cohesive ECM is populated with stem cells or resident cells to regenerate the organ [315]. The goal is to produce an organ with exquisite replication of phenotypic traits and cellular signal transduction to allow functional integration of the regenerated organ. Whole-organ bioengineering for the liver, heart, lungs, and kidneys is highly challenging because of its very high structural and functional complexity. A detailed review of all of the technologies applicable to organ regeneration would be beyond the scope of this work [315] [316] [317],., Thus, only a conceptual overview has been provided.

## Current Ethical Issues in Stem Cell and Regenerative Medicine

As described before, stem cell research with has great promise for medical applications. However, there are still challenges regarding technical matters. Besides this, other issues, including ethical dilemmas, need to be addressed. hESCs have been most widely used for basic and clinical research so far and thus have drawn significant attention regarding their ethical use. Such cells are directly taken from human tissues during pregnancy and abortion. Other challenges include immune rejection and depletion of cell resources after exogenous transplanting [318].

To produce NTSCs, SCNT technology also faces challenges of ethical concerns, as well as substantial technology, facility, and finance requirements. SCNT technology first became controversial after Dolly in 1996, due to concerns that it might be applied to human cloning. In 2005, the United Nations declared a ban on “all forms of human cloning inasmuch as they are incompatible with human dignity and the protection of human life” [319]. Although human reproductive cloning is associated with serious ethical objections, some hope that the creation of a global governance framework based on knowledge sharing and shared feasibility testing via intergovernmental organizations and stakeholders can maximize benefits to humans while avoiding ethical concerns [319].

iPSCs have noteworthy attributes compared with NTSCs and ESCs, including obviation of ethical issues and lower risks of immune rejection, contamination, and infection, as well as the ability to obtain large quantities for precision and personalized medicine. Despite the challenges that remain for iPSC clinical development, the advantages of iPSCs engender optimism regarding their feasibility and clinical potential. The first successful clinical case study of transplanting iPSC-derived retinal cells represents an advancement in stem cell-based regenerative medicine that avoided ethical concerns.

Recently, in 2018, human germline gene editing without authorization has revived controversy and debate. The Second International Summit on Human Gene Editing in Hong Kong made an announcement that a team successfully carried out the human germline gene editing by using CRISPR technologies to create gene-edited twins to “prevent” HIV infection [the website of The Second International Summit on Human Gene Editing]. This resulted in significant ethical complaints worldwide. Gene editing in human embryos to prevent or correct diseases holds significant potential; however, the current immature status of this technology without strict regulation is dangerous to society. Thus, future research in this field will neeed to account for governmental, society, and scientific approval and permission.

## Summary

Stem cell technologies represent a breakthrough development in biomedical science. There is optimism that iPSC technologies in particular may provide cures for human diseases because they enable cells from adult tissues to be reprogrammed into an embryonic-like state, thereby avoiding the ethical issues associated with ESCs. The potential applications of iPSCs have been expanded by state-of-the-art CRISPR/Cas9 genetic alterations, biomaterials, and 3D printing. hiPSCs have the potential to be developed into a wide range of specific subtypes of cells and, when combined with tissue engineering, they can even develop into tissues and organs. hiPSCs and other stem cells may someday be used to deliver personalized therapies.

In selecting an iPSC reprogramming method, it is important to minimize risk while maintaining pluripotency and the ability to direct specific cell fate development. As we have discussed, significant variation exists between reprogramming methods, each with their own advantages and disadvantages. For example, non-integrating, self-excising, and non-viral vectors are more clinically applicable at present than integrating vectors (Fig. 1 and 2). Non-viral vectors enable transcription factor gene delivery without anomalous genetic modifications. *Oct4* is the most important reprogramming gene, whereas *Klf4* and *c-Myc* can be replaced with *Nanog* and *Lin28*. Of the various genetic factors, signaling molecules, small molecules, microRNAs, and chemicals used to enhance reprogramming efficiency, we favor the CHALP molecule cocktail by Yu’s protocol in 2011 [102] or the most recent 6-small-molecule cocktail by Li’s protocol in 2016 [150]. Harvesting stem cells from urine samples represents an attractive noninvasive means of obtaining cells for reprogramming. Along these same lines, stem cells have now been sustained for long periods of time in a non-differentiating state, after which they can be differentiated in xeno-free media. Similarly, progress has been made regarding high-throughput, scalable expansion and controlled differentiation in a bioreactor culture system for large-scale studies, cell therapy, and industrial applications.

There are numerous technical and scientific challenges that need to be addressed before iPSC technology can be applied in a clinical setting. Beyond the primary concern of patient safety, there remains a need for consistent quality control and streamlined differentiation protocols and biomaterials for the translation of iPSCs into clinical applications [Fig. 1 and 2]. The field of stem cell and regenerative medicine is tremendously exciting and has the potential to someday revolutionize basic and clinical biomedical science.

## Abbreviations

3D: Three-dimensional
AST: Asterias Biotherapeutic
AZA: 5′ azacytidine
BMP: Bone morphological protein
Cas: CRISPR-associated gene
cGMP: Current good manufacturing practice
CRISPR: Clustered regularly interspaced short palindromic repeats
ECM: Extracellular matrix
Epi: *Episomal*
EPCs: Endothelial progenitor cells
ERK: Extracellular signal-regulated kinase
ESC: Embryonic stem cell
FGF: Fibroblast growth factor
GSK3: Glycogen synthase kinase-3
hESC: Human embryonic stem cell
HSCs: hematopoietic stem cells
iPSC: induced pluripotent stem cell
iNPCs: induced neural progenitor cells
hiPSC: Human induced (primed) pluripotent stem cell
hUCs: Human urine-derived cells
LIF: Leukemia inhibitory factor
MAPK: Mitogen-activated protein kinase
*MEK*: MAPK/ERK kinase
MEF: Mouse embryonic fibroblast
MSC: Mesenchymal stem cell
NCBI: National Center for Biotechnology Information
NPs: Neural progenitors
NTSCs: Nuclear transfer stem cells
OPC: Oligodendrocyte progenitor cell
OSKM: *Oct4*, *Sox2*, *Klf4*, and *c-Myc*, Yamanaka factors
PD: Parkinson disease
PSCs: Pluripotent stem cells
ROS: Reactive oxygen species
RSCs: Reprogramming Stem Cells
SCI: Spinal cord injury
SCNT: Somatic cell nuclear transfer (technique)
*SeV*: *Sendai-viral*
shRNA: short hairpin RNA
TCSCs: Tissue-committed stem cells
TGF: Transforming growth factor
TP53: Tumor protein p53, p53
UTR: Untranslated region
VSELs: Very Small Embryonic-Like Stem Cells
